# Classifying mild traumatic brain injuries with functional network analysis

**DOI:** 10.1186/s12918-018-0645-z

**Published:** 2018-12-21

**Authors:** F. Anthony San Lucas, John Redell, Dash Pramod, Yin Liu

**Affiliations:** 1 Department of Epidemiology, University of Texas M.D. Anderson Cancer Center, 1155 Pressler Street, Houston, TX USA; 2 Department of Neurobiology and Anatomy, University of Texas Health Science Center at Houston, 6431 Fannin Street, Houston, TX USA; 3 University of Texas Graduate School of Biomedical Science, 6767 Bertner Avenue, Houston, TX USA; 4 Center for Precision Health, School of Biomedical Informatics, The University of Texas Health Science Center at Houston, 7000 Fannin Street, Houston, TX USA

**Keywords:** mTBI subtype classification, Biomarkers, Weighted protein interaction network, Subnetwork modularity, Gene ontology annotation

## Abstract

**Background:**

Traumatic brain injury (TBI) represents a critical health problem of which timely diagnosis and treatment remain challenging. TBI is a result of an external force damaging brain tissue, accompanied by delayed pathogenic events which aggravate the injury. Molecular responses to different mild TBI subtypes have not been well characterized. TBI subtype classification is an important step towards the development and application of novel treatments. The computational systems biology approach is proved to be a promising tool in biomarker discovery for central nervous system injury.

**Results:**

In this study, we have performed a network-based analysis on gene expression profiles to identify functional gene subnetworks. The gene expression profiles were obtained from two experimental models of injury in rats: the controlled cortical impact and the fluid percussion injury. Our method integrates protein interaction information with gene expression profiles to identify subnetworks of genes as biomarkers. We have demonstrated that the selected gene subnetworks are more accurate to classify the heterogeneous responses to different injury models, compared to conventional analysis using individual marker genes selected without network information.

**Conclusions:**

The systems approach can lead to a better understanding of the underlying complexities of the molecular responses after TBI and the identified subnetworks will have important prognostic functions for patients who sustain mild TBIs.

## Background

Traumatic brain injury (TBI) results from an external force causing immediate damage to brain tissue, followed by secondary pathogenic events which ultimately give rise to neurodegeneration. Dependent on the context of the primary injury, different cell responses are initiated, which can exacerbate the injury to varying degrees. Cell death resulting from the initial impact on the brain tissue is irreversible, so treatments normally focus on minimizing the secondary injury that is due to these cell responses [1]. To date, these secondary injury responses have been poorly characterized, leaving molecular classification of TBI difficult [2, 3]. TBI remains a leading cause of death and disability in the industrialized countries and represents a growing health problem [4]. Thus, even a modest improvement in patient outcome could have significant public health benefits [5, 6]. It is estimated that at least 25% of patients experiencing a mild TBI (mTBI) do not seek hospital care [7]. Among these mTBI patients, some of the post-concussive symptoms have been reported to remain up to one year or more and can significantly affect the long-term morbidities [8]. It has been shown that concussive force can elicit physical and structural changes in the brain. These changes can be focal or diffuse through the brain [9]. Therefore, identification of both common and pathology-specific molecular mechanisms underlying different types of injuries may aid in identification of targets for effective TBI treatments. We have utilized two common experimental models of injury in this study: the mild controlled cortical impact (mCCI) model that causes a focal injury, and the mild fluid percussion injury (mFPI) model that causes a more diffuse brain injury. Both injury models qualitatively recapitulate a number of functional deficits and pathological responses exhibited in human TBI cases. In this study, we employ a systems approach to improving the identification of biomarkers that can distinguish there two models. These biomarkers, if successfully identified, could be used to better guide treatments to mTBI patients, and more optimistically they could be potential targets of novel treatments.

Recent years have witnessed an increasing number of disease markers identified through computational analysis of genome-wide expression profiles. Typically, gene expression profiling studies are limited to focus on individual genes that are significantly differentially expressed between different classes of diseases. However, single-gene analyses have been criticized for several reasons [10, 11]. In the cases of mTBI classification, if we only examined the differences in the expression levels of individual genes across different mTBI models and neglected the genes that are not associated with a TBI subtype at a significance threshold, we would fail to account for the complexities and redundancies that arise from gene interactions inherent to the mTBI responses. Discarded genes showing modest differential expression between mTBI classes may represent important biomarkers of mTBI. In this study, we have proposed a data-driven model and identified biomarkers not as individual genes but as gene subnetworks, by incorporating the gene expression profiles from injury models and the protein-protein interaction information from existing databases. The genes in each of the identified subnetworks are expected to be highly correlated with each other and exhibit a coherent expression profile across samples, while others exist as background noise. It is also expected that the genes in a functional subnetwork exhibit high topological similarity with each other and should lead to a biologically meaningful sample classification. The network-based approach has been widely adopted to identify gene subnetworks as biomarkers in the field of cancer research and other human diseases [12, 13], but has never been applied in the discovery of biomarkers for brain injury. Here, with simulation and real data analysis, we have demonstrated that our computational systems approach based on network theory performs better than individual gene analyses as well as other gene grouping strategies in mTBI classification. The identified subnetworks can provide insights into the multifactorial relationships of genes and delineate the underlying complexities of the biological processes involved in different mTBI classes.

## Methods

### Animal subjects and surgeries

Male Sprague-Dawley (SD) rats (275–300 g) were purchased from Charles River Laboratories (Wilmington, MA). All experimental procedures were approved by the local Institutional Animal Care and Use Committee and were conducted according to the recommendations provided in the Guide for the Care and Use of Laboratory Animals. Protocols were designed to minimize pain and discomfort during the injury procedure and recovery period. Our mCCI and mFPI injury models were described in [3]. After injury preparation, animals were placed in a warm chamber and allowed to completely recover from anesthesia, and then returned to their home cages.

### Gene expression microarray

Using the mTBI animals (*n* = 4/group), ipsilateral cortical issues underlying the injury site was quickly dissected at 24 h post-injury. Total RNA from the cortical tissue was isolated using the mirVana miRNA Isolation Kit (Invitrogen, Carlsbad, CA), following the manufacturers’ recommended protocol, and amplified using the Illumina TotalPrep RNA Amplification Kit (Ambion, Austin, TX). RNA amplification and microarray hybridization were carried out by The University of Texas Health Science Center Houston Microarray Core Laboratory (Houston, TX). Briefly, first-strand complementary DNA (cDNA) was generated from total RNA by reverse transcription. Second strand cDNA synthesis was initiated by the addition of RNase H/DNA polymerase mix. The complementary RNA (cRNA) was amplified by the in vitro transcription reaction (IVT). cRNA (750 ng) was loaded onto RatRefSeq-12 Illumina Sentrix Beadchip Arrays (Illumina, Inc., San Diego, CA), hybridized overnight, washed, and incubated with streptavidin-Cy3 to detect hybridized biotin-labeled cRNA probes. Arrays were dried and scanned with a BeadArray Reader (Illumina). It was noted that most raw gene expression values were not normally distributed but highly skewed. Therefore, the Box-Cox transformation [14] was used to normalize the distribution for each gene expression values. The Kolmogorov-Smirnov test was used to test for normality of the transformed distribution at a 5% significance level.

### Constructing a weighted network from protein interaction information and gene expression data

Experimentally detected protein-protein interactions (PPIs) were downloaded from BioGRID [15], DIP [16], and HPRD [17] databases. Since there are a limited number of experiments detecting PPIs in the rat genome, we also obtained predicted rat PPIs based on onthology, where orthologous interactions were generated by mapping experimentally detected PPIs in human or mouse genomes to pairs of orthologs in rat genome, if such orthologs are available in HomoloGene database [18]. Each edge of the protein interaction network was further weighted by overlaying gene expression information. Specifically, we calculated the absolute value of the Pearson correlation coefficient abs(cor(x_i_,x_j_)) as the edge weight, where *x*_*i*_ and *x*_*j*_ represented the normalized gene expression vectors for genes *i* and *j*, respectively. Therefore, each edge of the protein interaction network was weighted by the level of co-expression between its two corresponding genes, with the weights between 0 and 1.

### Identifying significant subnetworks

We performed functional network analysis by following the protocol described in [19]. We defined the subnetwork scoring function *S* as a weighted sum of class relevance R and modularity M.

1 $$ \mathrm{S}=\beta \mathrm{M}+\mathrm{R} $$

Here *M* describes the subnetwork connectivity, and *R* is a measure of the discriminatory power of the subnetwork genes to differentiate classes. In addition, the parameter *β* allows us to trade off the effects of the gene expression information with the network modularity on the subnetwork score. To simplify the scoring algorithm, we set β = 1, assuming equal weights of network modularity and class relevance on calculating the network score.

To get a measure of how strongly the genes within a subnetwork are connected, the modularity *M* was calculated as the mean clustering coefficients *C*_*i*_ of the genes in a subnetwork,

2 $$ \mathrm{M}=\frac{\sum_{\mathrm{i}}{\mathrm{C}}_{\mathrm{i}}}{\mathrm{n}}\left[\mathrm{for}\ \mathrm{n}>=3;0\ \mathrm{otherwise}\right] $$

(3)| where *C*_*i*_ was defined as in Dong and Horvath [20, 21]. *C*_*i*_ is the clustering coefficient for node *i*, where nodes *l* and *m* are node neighbors of *i*, and *w* represents the weight of the edge between nodes in the subnetwork:

$$ {C}_i=\frac{\sum_{l\ne i}{\sum}_{m\ne i,m\ne l}{w}_{il}{w}_{lm}{w}_{mi}}{{\left({\sum}_{l\ne i}{w}_{il}\right)}^2-{\sum}_{l\ne i}{w}_{il}^2} $$

Intuitively, *C*_*i*_ is a ratio of the weighted triangles that can be made with node *i* and its neighbors over the sum of the weighted possible triangles extending off of node *i*. For a general weighted network with edge weights between 0 and 1, the clustering coefficient of node *i, C*_*i*_ also lies between 0 and 1. *C*_*i*_ equals 1 if and only if all neighbors of node *i* are connected to each other.

The class relevance *R* is a measure of the ability for a subnetwork to distinguish two classes. To calculate this, the expression values of each gene *i* in sample *j* were first normalized to z-scores, z_ij_, which had a mean of 0 and standard deviation of 1 for each gene over all samples. The individual z-scores of each member gene in the subnetwork *K* for sample *j* were averaged into a summarized expression $\mathrm{Vkj}=\frac{\sum_{i=1}^n{z}_{ij}}{n}$, where *n* is the number of genes in the subnetwork K. A t-test was then used to compare the summarized expression values of samples between two classes and the resulting t-value was denoted as the class relevance *R*. In this study, the two classes refer to mCCI and mFPI injury models.

**Fig. 1:**
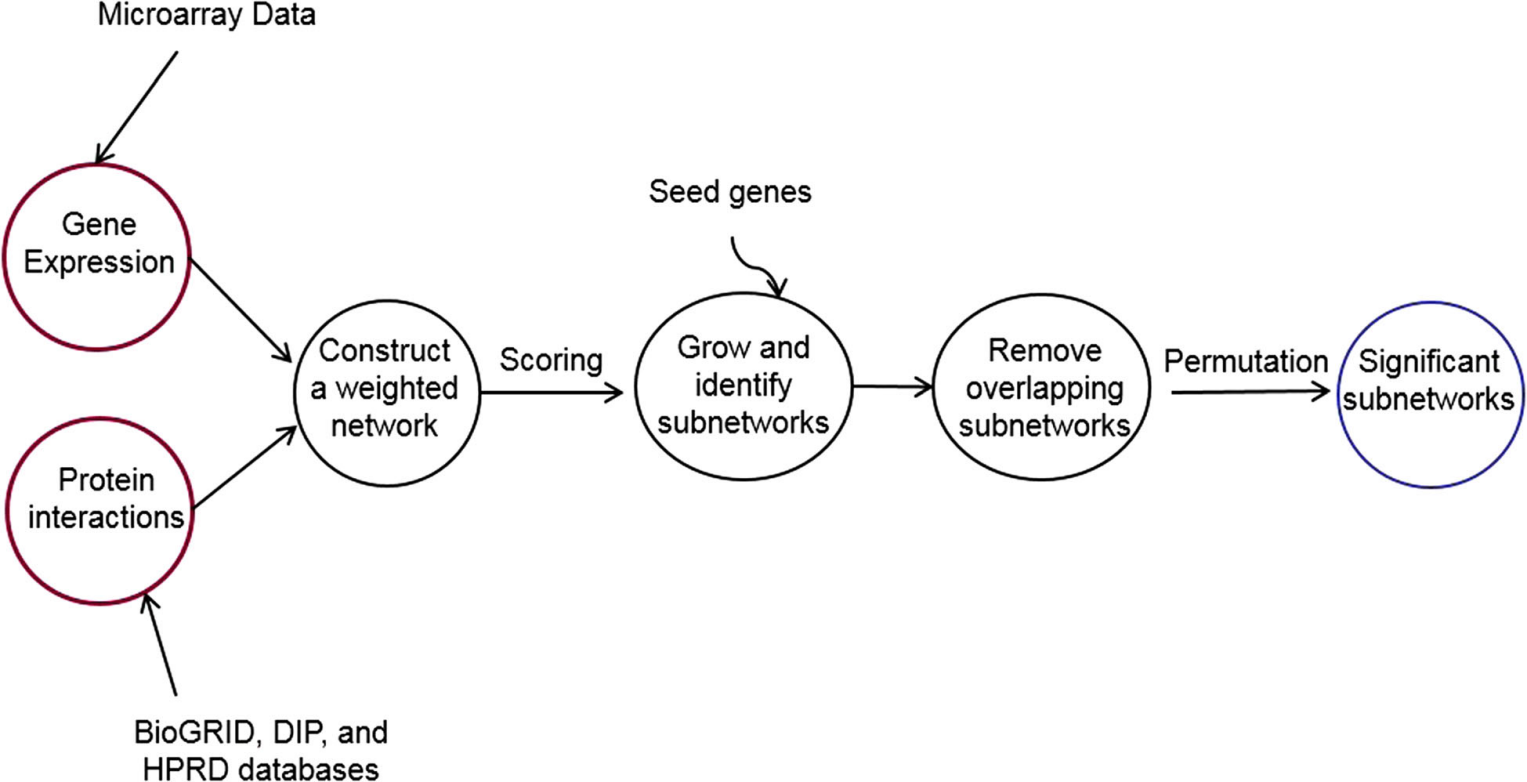
An overview of subnetwork identification approach

A framework demonstrating the steps for finding significant subnetworks is described in Fig. 1. We applied the greedy search algorithm in searching subnetworks [22]. To identify significant subnetworks that discriminate mCCI and mFPI, candidate subnetworks were scored comparing two classes. First, individual differentially expressed genes were used as seeds for growing potential subnetworks. For each seed, two neighboring genes were iteratively added to the seed and subnetwork scores were recalculated. The pair of neighboring genes that yielded the biggest improvement in subnetwork score was added to the seed to form an initial subnetwork of three genes (i.e., an initial triangular subnetwork). Single neighbor nodes were then added iteratively until the subnetwork score could no longer be improved. It is likely that genes are shared across different subnetworks, resulting in potentially redundant subnetworks. The redundant subnetworks were removed by the following steps:

1. Obtaining the scores of all subnetworks and sorting them in a descending order of scores.
2. Iterating through the list of subnetworks and checking for redundancy.

- if a subnetwork was contained within a higher-scoring subnetwork, we discarded the lower-scoring subnetwork;
- if a subnetwork was a super set of a higher-scoring subnetwork, we discarded the super set;
- if there was an overlap in genes between a lower-scoring and higher-scoring subnetwork:

i. if the overlap ≥50% (number of overlapping genes/total number of unique genes), we discarded lower-scoring subnetwork;
ii. if the overlap < 50%, we kept both subnetworks (document them for manual inspection).

To select the significant subnetworks, we calculated the empirical *p*-values of the identified subnetworks. We first generated the null distribution by permuting the expression vector of genes in the full network. This permutation test dissociated the relationship between protein interaction and gene expression information. We then ran the same subnetwork identification procedure on the permuted data. This process was repeated 100 times and the scores of the resulted random subnetworks were recorded for each permutation. The empirical adjusted *p*-value for the real subnetwork score was calculated as the fraction of the random subnetworks having a higher score than that real subnetwork [23]. Only the subnetworks with empirical adjusted *p*-values smaller than 0.05 were selected for further evaluation and analysis.

### Gene grouping strategies

To evaluate our approach for identifying biomarkers that distinguish different mTBI classes (mCCI vs. mFPI), we compared our approach with other gene grouping strategies to be used in classification. Two other gene grouping strategies were included in this study: 1) Pathway based gene sets using the list of canonical pathways extracted from the Molecular Signature Database (MSigDB) [24]. 2) Functionally related gene sets based on Gene Ontology (GO) annotations. GO gene sets were determined by retrieving genes for all GO terms that contained less than 50 genes, in order to eliminate the GO terms that were too general in function annotation. The resulted two groups of gene sets were evaluated for their discriminatory potential in classifying TBI classes. For each gene grouping strategy, expression values for each gene set were converted into summarized expression scores as described previously. These expression scores were used to test differential expression between mCCI and mFPI classes and gene sets were ranked according to their discriminatory powers. After redundant gene sets were removed, the resulting gene sets were used as features in training algorithms to build models for predicting TBI classes.

## Results

### Protein interaction network weighted with gene co-expression data

**Fig. 2:**
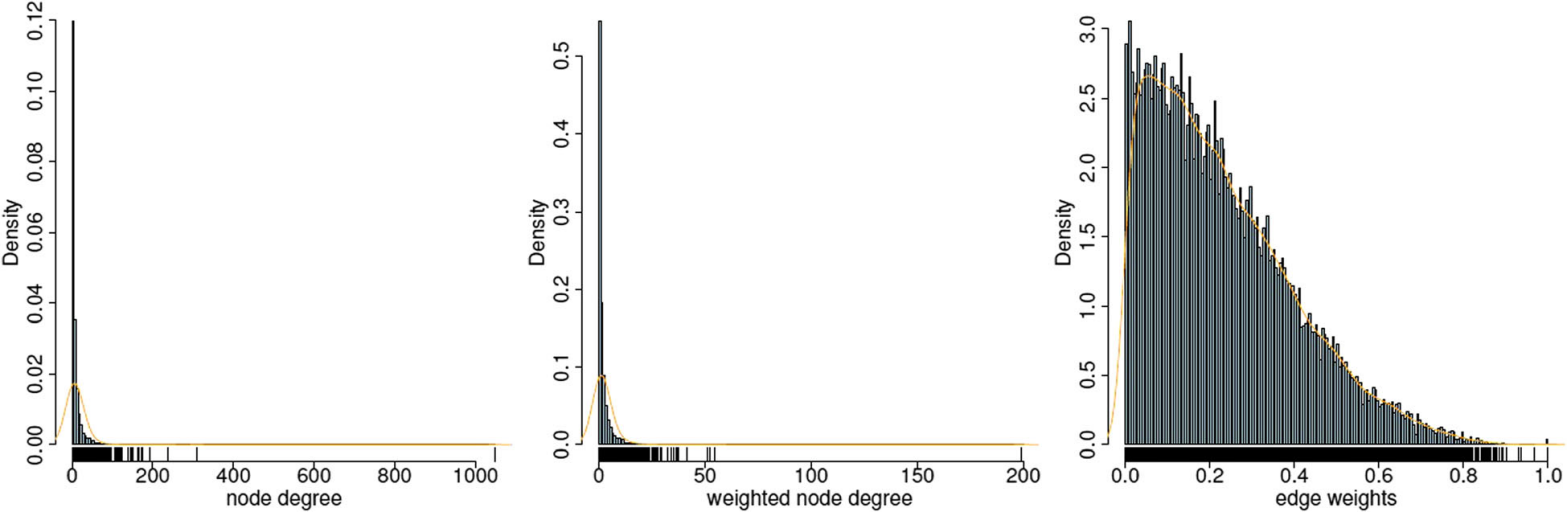
Network structure profile. Distribution of node degrees in PPI, weighted node degrees and edge weights in the constructed weighted network. The y-axis represents the probability density for each of the distribution

Given the resources of protein interactions as described in the Materials and Methods section, a protein interaction network was constructed with 18,781 proteins and 207,829 edges. Gene expression values were then overlaid on the protein interaction network. Each edge of this subnetwork was weighted by the level of co-expression between its two corresponding genes using Pearson correlation, as described previously. Fig. 2 shows the distribution of node degrees, weighted node degrees and edge weights for this network. There were 3 outlier gene nodes that had over 200 direct neighbors. These genes can be problematic depending on how subnetworks are searched for. In our current method of finding subnetworks, we aimed to find complex interactions within a small set of genes, more specifically, we looked for enriched triangular relationships amongst genes. Since these particular hub genes could generate a lot of star-shaped structures for subnetworks, they were removed from the network subject to further network analysis.

### Overview of functional subnetworks

**Fig. 3:**
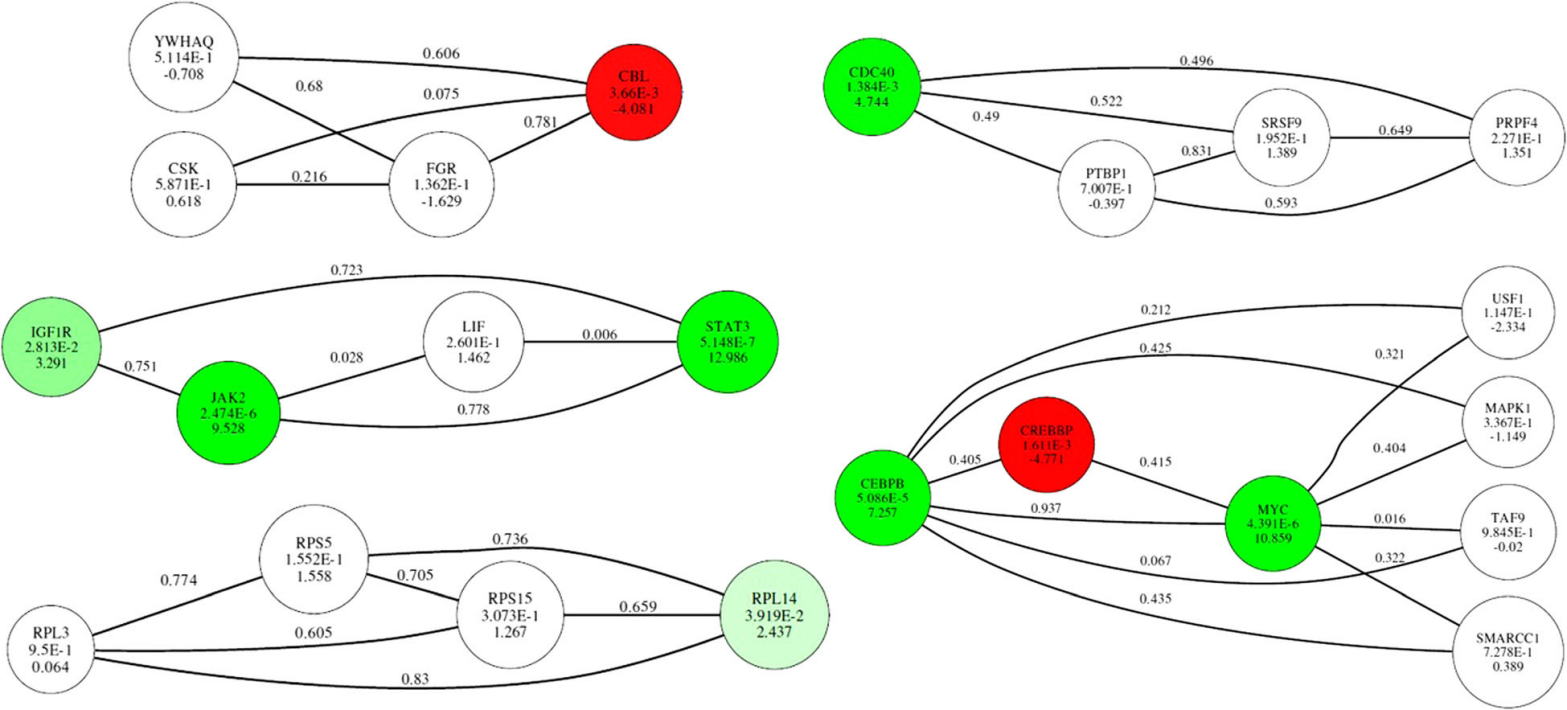
Top five most significant subnetworks. The colors on the nodes indicate how the gene node differentiates between the mCCI and mFPI classes. Green indicates that the expression level of the gene in the mCCI class is significantly higher compared to that in the mFPI class. Red indicates that the gene expression level in the mCCI class is significantly lower compared to that in the mFPI class. If a node is white, the corresponding gene does not significantly differentiate (*p*-value < 0.05) the two sample groups. The intensity of the color corresponds to the level of significance. The numbers inside each node represent the discriminatory power of the gene, indicated by the t-score and the corresponding p-value. The numbers along each edge represent the edge weights in our constructed weighted network

The subnetwork identification step yielded a total of 189 significant subnetworks, consisting of 695 genes. An example of the resulting discriminative subnetworks is shown in Fig. 3. The genes mitogen-activated-protein kinase 1 (MAPK1) and interleukin 6 family cytokine (LIF) did not show significant differentiated expression between mCCI and mFPI samples, but they played an important role in the subnetwork by interconnecting many differentially expressed genes, such as CEBPB, MYC, JAK2 and STAT3. Given the fact that both MPAK1 and LIF genes are well-known players in the cytokine signaling pathway involved in inflammatory response, our results suggest they can serve as potential targets for intervention. To further investigate the functions of the identified subnetworks, we extracted the Biological Process annotations from Gene Ontology (GO) database [25], and examined whether any GO terms were overrepresented by the union of genes in the 50 most significant subnetworks, compared to an expected genome-wide representation [26]. Because there is a lot of redundancy in the GO tree, we used the GO terms from levels 3 to 10 to determine specific biological process categories which the subnetwork genes belong to [27]. The GO enrichment *p*-values were calculated by the hyper-geometric test, followed by Benjamini and Hochberg’s multiple hypotheses testing correction procedure [28]. The ten most significantly enriched GO terms are listed in Table 1. Overall, the GO analysis of the top 50 subnetworks showed significant overrepresentation of genes belonging to some fundamental cellular processes, such as cell differentiation and cell-cell adhesion. We also found there were significant enrichment of GO terms related to brain injury, such as neuron development, neurogenesis, ion membrane transport, and blood coagulation.

**Table 1:** Gene Ontology (GO) biological process annotations for significant subnetwork genes

GO Biological Process	FDR
Dendrite development	8.16E-08
Neuron development	2.86E-07
Regulation of cell differentiation	8.11E-07
Neurogenesis	5.41E-06
Regulation of programmed cell death	8.19E-06
Regulation of membrane potential	1.02E-05
Ion transmembrane transport	1.13E-05
Cell-cell adhesion	1.68E-05
Blood coagulation	4.52 E-05
Wnt signaling pathway	6.32E-04

The top 10 most enriched GO biological process terms with their corresponding corrected *p*-values are listed. FDR, adjusted *p*-values for multiple testing by Benjamini and Hochberg’s procedure

### mTBI subtype classification evaluation

**Fig. 4:**
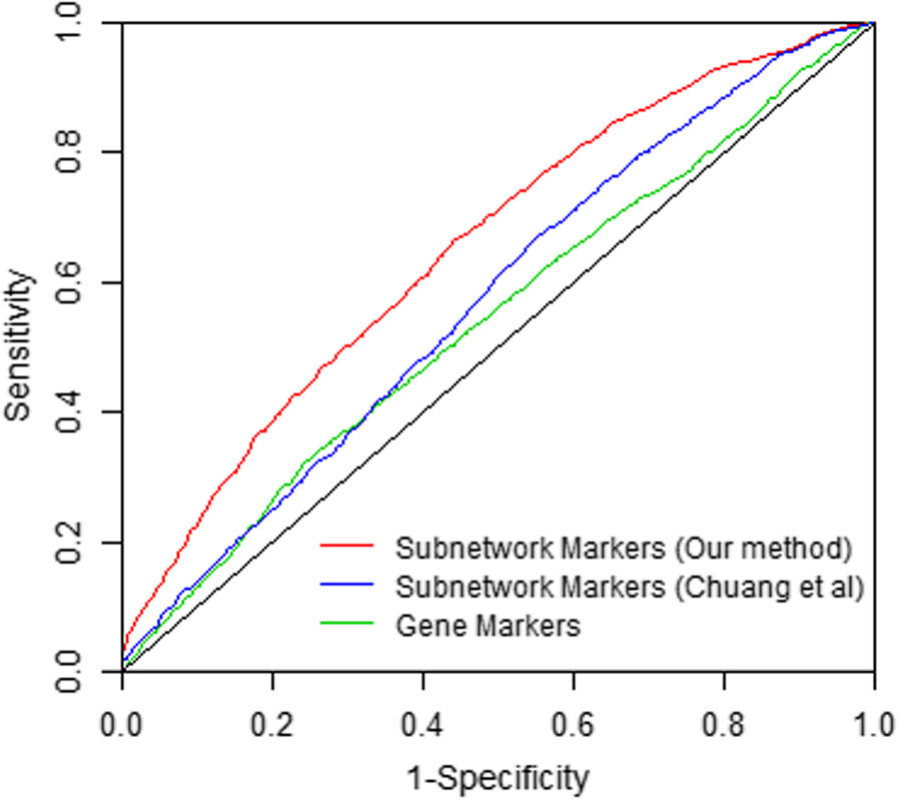
Comparison of classification performance between subnetwork markers and individual genes. The activities of identified subnetworks, calculated as the mean activities of its member genes, are used as the features of the support vector machine (SVM) to classify the samples. The classifier performance is measured by ROC curves

Given the identified subnetworks, we tested their validity and performance in the classification problem. However, in this study, we only had experimental data available for 8 rats (4 samples/injury model). The small sample size made it difficult to train and test a classifier. Therefore, we performed a simulation study to achieve an unbiased classification evaluation. The mean and standard deviation of each gene were estimated from the observed data. Given these parameters, we used the packages in R studio [29] to simulate gene expression datasets corresponding to mCCI and mFPI classes, with 100 samples per class. Using the simulated datasets, we performed a five-fold cross validation to compute the classification accuracy. First, the simulated gene expression data corresponding to the identified subnetwork markers were used to encode features for a Support Vector Machine (SVM) classifier [30]. Then, we divided the mCCI and mFPI samples into five equal parts, respectively. We used four-fifth of the samples to train SVM and the remaining one-fifth of samples to test the trained classifier. Finally, we evaluated the sensitivity and specificity of our method and calculated its ROC curves and the areas under ROC curve (AUC). We compared the performance of TBI subtype classification of our subnetwork markers with the genes that were most significantly differentially expressed between mCCI and mFPI samples. We selected the top 695 individual genes, the same number of genes as that in the union of identified subnetworks, to achieve a fair comparison. Our subnetworks yielded an AUC score of 0.71, while the individual gene set yielded a lower AUC score of 0.58 (Fig. 4). Therefore, the subnetwork markers identified based on our network analysis outperformed the individual gene markers in classifying mild TBI subtypes. In addition, we compared our method with another subnetwork identification method by Chuang et al.,, which didn’t consider the subnetwork modularity when identifying subnetworks [13]. As a result, it led to many star-shaped networks. When comparing the method performance using ROC curves, we found our method performed better than the method by Chuang et al. [13], based on the comparison of sensitivity and specificity (Fig. 4).

**Fig. 5:**
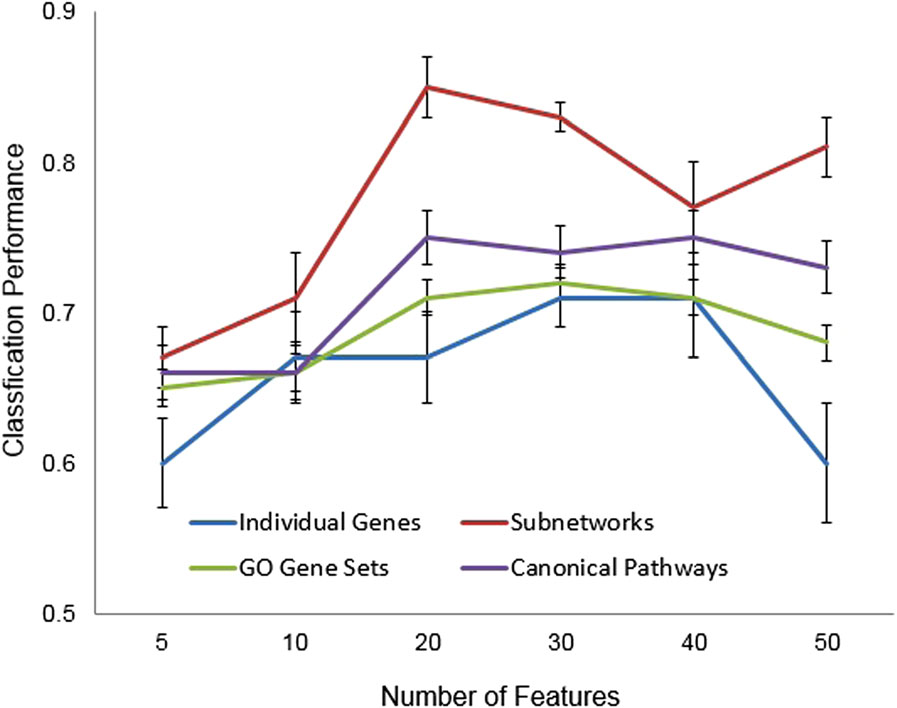
Comparison of classification performance among subnetworks, individual genes, and predefined functionally gene sets from GO or MSigDB. For each size of feature set, five iterations of five-fold cross validation are used to split the dataset, train, and evaluate classifier. The curves show the median of classification performance, measured by the F-scores, and error bars indicate the standard deviation over five cross-validation experiments

We also compared the performance of TBI subtype classification of the subnetwork markers with those based on predefined functionally related genes extracted from GO annotation and canonical pathways. The classification results were evaluated using an F-score as in [31], where F = 2* Precision*Recall/(Precision + Recall). In this particular classification task, the precision is the proportion of classified mCCI samples that are true mCCIs, and the recall is the proportion of true mCCI samples that are correctly classified by a method. Similarly, we performed a five-fold cross validation to compute the classification accuracy. Using the features drawn from different gene sets, we trained SVM based on the simulated gene expression data from four-fifth of samples, and then we tested the performance of the learned feature weights on the remaining one-fifth of samples. We repeated the process for five times to obtain an averaged F-score over iterations of cross validation experiments. We examined the classification accuracy using different sizes of feature set (the top 5, 10, 20, 30, 40, or 50 features), and summarized the comparison results in Fig. 5. It is demonstrated that over all the tests, the SVM using 20 functional subnetwork features achieves the highest performance with an F-score of 0.85. We have also shown the functional subnetworks outperform significant individual genes or predefined functionally related genes across different sizes of feature sets, indicating the advantage of using gene subnetworks for sample classification and prediction.

## Discussion

The parameter setting in variable selection methods can impact the performance of the selected feature genes sets. In this study, we showed the relative robustness and superior performance of our network analysis across different numbers of selected features. We have demonstrated that effectively incorporating gene expression profiles into protein interaction information can identify functional subnetworks that better classify different classes of mTBI than the gene markers selected without network information. We understand that translating the knowledge gained from an animal model onto molecular biomarkers identification in patients is practically challenging, simply because the brain tissue in TBI patients is rarely available, but the use of peripheral tissues such as lymphoblast or blood could be a potential solution.

## Conclusions

We have aimed to improve the identification of biomarkers that can distinguish two different classes of TBI in rodent animal models: the mild Controlled Cortical Impact (mCCI) and the mild Fluid Percussion Injury (mFPI), representing focal and diffuse TBIs, respectively. We have developed and applied a network-based approach on gene expression profiles from the entire rat genome. Our network based analysis can identify genes that are essential for maintaining the integrity of a subnetwork whose overall expression is discriminative between samples. In addition, we demonstrated that our network-based analysis achieves higher sensitivity and specificity in differentiating the heterogeneous responses corresponding to different classes of mTBI, compared to conventional analyses using either individual genes or predefined functionally related gene sets. These identified biomarkers could be used to better direct the diagnosis and treatment to TBI patients, and more optimistically, they could help to develop rationale-based therapies for treating the millions of Americans who suffer from TBI.

## Abbreviations

AUC: The areas under a receiver operating characteristic curve
GO: Gene ontology
KEGG: Kyoto encyclopedia of genes and genomes
mCCI: Mild controlled cortical impact
mFPI: Mild fluid percussion injury
MSigDB: Molecular signature database
PPI: Protein-protein interaction
ROC curve: Receiver operating characteristic curve
SVM: Support vector machine
TBI: Traumatic brain injury
